# Patients’ experiences of breathing retraining for asthma: a qualitative process analysis of participants in the intervention arms of the BREATHE trial

**DOI:** 10.1038/s41533-017-0055-5

**Published:** 2017-10-05

**Authors:** Emily Arden-Close, Lucy Yardley, Sarah Kirby, Mike Thomas, Anne Bruton

**Affiliations:** 1 Department of Psychology, Bournemouth University, Bournemouth, UK; 2 Academic Unit of Psychology, University of Southampton, Southampton, UK; 3 NIHR Southampton Respiratory Biomedical Research Unit, University of Southampton, Southampton, UK; 4 Primary Care and Population Sciences, University of Southampton, Southampton, UK; 5 Faculty of Health Sciences, University of Southampton, Southampton, UK

## Abstract

Poor symptom control and impaired quality of life are common in adults with asthma, and breathing retraining exercises may be an effective method of self-management. This study aimed to explore the experiences of participants in the intervention arms of the BREATHE trial, which investigated the effectiveness of breathing retraining as a mode of asthma management. Sixteen people with asthma (11 women, 8 per group) who had taken part in the intervention arms of the BREATHE trial (breathing retraining delivered by digital versatile disc (DVD) or face-to-face sessions with a respiratory physiotherapist) took part in semi-structured telephone interviews about their experiences. Interviews were analysed using thematic analysis. Breathing retraining was perceived positively as a method of asthma management. Motivations for taking part included being asked, to enhance progress in research, to feel better/reduce symptoms, and to reduce medication. Participants were positive about the physiotherapist, liked having the materials tailored, found meetings motivational, and liked the DVD and booklet. The impact of breathing retraining following regular practice included increased awareness of breathing and development of new habits. Benefits of breathing retraining included increased control over breathing, reduced need for medication, feeling more relaxed, and improved health and quality of life. Problems included finding time to practice the exercises, and difficulty mastering techniques. Breathing retraining was acceptable and valued by almost all participants, and many reported improved wellbeing. Face to face physiotherapy was well received. However, some participants in the DVD group mentioned being unable to master techniques.

## Asthma: Patients receptive to breathing retraining

Patients with asthma taught how to change their unconscious breathing patterns generally like non-pharmacological interventions. Researchers in the UK, led by Mike Thomas from the University of Southampton, interviewed 16 people about their experiences in a trial that tested breathing retraining exercises delivered by DVD or face-to-face sessions with a respiratory physiotherapist. Overwhelmingly, trial participants reported that breathing retraining sessions gave them greater control over their symptoms, helped them relax, improved their quality of life and reduced the need for medications. Some participants who received DVD instruction said they had trouble mastering the techniques, and many in both groups found it hard to find time to practice the exercises. Overall, however, patients were positive about the experience. The authors conclude that breathing exercises are likely to be a well-received method of asthma management.

## Introduction

Asthma affects over five million people in the UK.^1^ It is typically managed pharmacologically by inhaled or oral medications (including β_2_-adrenergic agonists and inhaled corticosteroids) or injections. However, fewer than 50% of adults with asthma achieve good symptom control.^2,3^ Many have tried non-pharmacological treatments, which they feel may improve general health^4^ and reduce the need for pharmacological treatment.^5^ In particular, breathing retraining has recently increased in popularity due to greater awareness and accessibility of asthma-orientated breathing modification techniques (e.g., physiotherapist-taught programmes, the Buteyko breathing technique, and hatha yoga).^6–11^

Breathing retraining involves teaching breathing techniques (e.g., advice on route of breathing, slow breathing, relaxation techniques) to modify breathing patterns and improve breathing efficiency.^8^ It can lead to reduced symptoms and improved quality of life (QoL) for people with asthma, may reduce use of reliever medication,^12^ and has been recommended as an evidence-based adjuvant treatment for adults whose asthma remains uncontrolled despite standard treatments.^13^ A think-aloud study suggested breathing retraining exercises were acceptable in principle due to being non-pharmacological, holistic, and unobtrusive, and were likely to increase confidence in managing symptoms.^14^

Breathing retraining may therefore be an effective adjunct to medication, particularly for patients with poor asthma control. However, currently services require patients to be referred to respiratory physiotherapists who have undertaken specialist postgraduate training, which may have limited availability. Consequently, less resource intensive modes of delivery such as written or digitally recorded audio-visual self-help programmes may provide an acceptable alternative for those willing to try an instantly available alternative treatment. Instructional videos to deliver breathing retraining reduced reliever inhaler use in an Australian study.^15^

This study was nested within the trial BREATHE (Breathing REtraining for Asthma Trial of Home Exercises; registered at www.ISRCTN.org, registration 88318003),^16^ which compared the effectiveness of breathing retraining delivered by digital versatile disc (DVD) with printed support materials with face-to-face physiotherapy and usual care. Including qualitative work in clinical trials helps facilitate understanding of participants’ experiences and acceptability of therapy.^17^ No previous research has assessed participants’ experiences of breathing retraining in practice. This study aimed to explore the experiences of participants in the intervention arms (DVD and face-to-face physiotherapy) of the BREATHE trial.

## Results

**Fig. 1:**
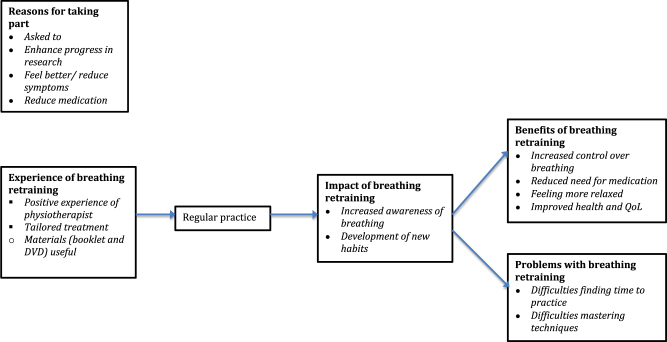
Themes and subthemes identified

Interviews lasted for 20 min on average (range 10 to 45 min). Five main themes emerged, which are presented in Fig. 1: *reasons for taking part, experience of breathing retraining, impact of breathing retraining, benefits of breathing retraining and problems with breathing retraining*.

### Reasons for taking part

Participants took part *because they were asked to, to enhance progress in research, feel better/reduce symptoms*, and *reduce medication*. Some took part because a healthcare professional asked them:

“I had my um annual asthma check-up and they just asked me if I would do it kind of there and then so I just said that I would.“ (P9, female, DVD)

Some felt it was their duty to *enhance progress in research* by giving something back to help enhance knowledge.

*“*because I would support research that is going to improve things for human beings.” (P16, male, F2F)

Many felt it would help *improve their health and symptoms*.

*“*I was just hoping it would … help my breathing when I went up hills … because that’s what I was particularly concerned with.” (P2, female, DVD)

Related to this, many participants wanted to *reduce their medication*. While they took it as required, they wanted to be less dependent on it.

*“*I liked the idea of a natural solution to the asthma rather than having to take medication.” (P4, female, F2F)

### Experience of breathing retraining

The participants in the face-to-face group had a *positive experience of the physiotherapist*, who *tailored the treatment* to their needs, and found sessions *motivational*. The *materials (booklet and DVD)* were also *considered useful*.

All the participants in the face-to-face group had a *positive experience of the physiotherapist* as friendly, helpful, supportive and patient:

*“*the lady that did them was really, really nice. She wasn’t condescending in any way, she was really patient, she was very quick to praise when you did it right” (P14, female, F2F)

Participants also said the physiotherapist *tailored the treatment* to their individual needs. When they were experiencing difficulties, she reviewed the techniques, and helped to break down goals into more manageable ones. For example, one participant described this experience of improving nose breathing:

“When I spoke to [the physiotherapist] about it, originally because I was having problems with it, she said just try and set myself little goals. So what I do is when I leave the house I set myself a goal to breathe through my nose to a certain point and then I will do it again, you know, I will get to this point and then I will try and breathe through my nose again.” (P5, female, F2F)

Tailored support from the physiotherapist also facilitated mastery of the techniques:

*“* … when she said, “Now, I need you to do these exercises at home,” she saw the look on my face and she said, “Would you like me to write the instructions down?” And I said, “Yes, please.” And, because I’d already done the actual training bit in my session the instructions put it back into my mind what I had to do and I found it really, really informative.” (P14, female, F2F)

The face-to-face group also found seeing the physiotherapist *motivational*. Knowing they would attend appointments prompted them to prioritize practising breathing techniques.

“I liked having the person there. It is not so much that she told me off when I hadn’t done the exercises but it is like an extra conscience.” (P5, female, F2F)

The *materials (booklet and DVD)* were also considered useful. Participants commented that both the booklet and DVD were helpful when practising, and the booklet also reminded them to do their exercises, and enabled them to log results. Some preferred the DVD because it showed them how to do the exercises,

“… with the DVD it was actually showing you.” (P11, male, DVD),

whereas some preferred the booklet, because it could be carried anywhere.

“I liked the booklet better … because I could just pick it up and, you know, look at it and do some of the exercises when I wanted to.” (P2, female, DVD)

Generally, participants felt the booklet and DVD complemented each other.

*“*I found by reading the booklet and then watching the DVD the two matched and I could see what was meant.” (P13, female, DVD)

### Impact of breathing retraining

Participants felt that breathing retraining led to *increased awareness of breathing*, and the *development of new habits*. Many participants reported initially practising breathing techniques *regularly* (more than three times a day), in line with recommendations, which they felt had facilitated development of new habits. Also, many mentioned increased *awareness of breathing*. Talking to the physiotherapist or watching the DVD and practising the exercises made them aware they had been breathing incorrectly.

“I’m a habitual mouth breather and to realise that I’d been breathing wrong all my life was a bit of an eye opener”. (P10, female, F2F)

“I do try to make myself aware of breathing through my nose all the time.” (P11, male, DVD)

Many participants mentioned being able to do stomach breathing and nose breathing automatically. They had internalised this new way of breathing so it became a *habit*:

“I can at rest actually do the stomach breathing pretty much naturally now.” (P3, female, DVD)

“I still try and do it [nose breathing].” (P12, male, F2F)

### Benefits of breathing retraining

Participants mentioned many health benefits they associated with breathing retraining, including *increased control over breathing, reduced need for medication, feeling more relaxed*, and *improved health and QoL*. Almost all participants mentioned *increased control over breathing*, including being able to use the techniques to breathe through asthma attacks.

“I had two asthma attacks last year … and actually being able to do this breathing helped a lot and I didn’t have to go to hospital. “ (P14, female, F2F)

Related to this, breathing retraining was often associated with *reduced need for medication*. Many participants reported using breathing techniques rather than reaching for reliever inhalers when they felt symptoms coming on.

“I don’t have to keep getting my inhaler and taking my inhaler, I can literally just do some of these breathing and I feel much better.” (P5, female, F2F)

Participants also mentioned that the breathing techniques *helped them to relax*.

“… when things have got a bit busy I have been very conscious to do it and I’ve found it very helpful and very calming.” (P15, male, F2F)

Other benefits attributed to breathing retraining under the umbrella of *improved health and QoL* included being less wheezy, sleeping better and having more energy

“I sleep so much better.” (P14, female, F2F)

“I also used to get very wheezy first thing in the morning and that doesn’t seem to be happening now.”(P3, female, DVD)

### Problems with breathing retraining

Participants also mentioned *problems with breathing retraining*. These included *difficulties finding time to practice* and *mastering techniques*. Many participants said it was difficult to *find time to practice* the breathing techniques before they fitted into daily routines.

“Initially quite hard to get started. It was finding the time I think and putting aside a regular time so that I didn’t skip things.” (P13, female, DVD)

Barriers included busy schedules and difficulties trying to fit it in during the daytime, meaning high motivation was required to carry it out:

“I could do as many BREATHE’s in the evening when I’m sat at home… but finding the time throughout the day when I’m at work, that was a bit more challenging.” (P5, female, F2F)

Participants also mentioned *difficulties mastering techniques*. Many participants found breath holding most difficult:

“the most difficult I found holding my breath, “ (P4, female, face-to-face)

Ease of mastering techniques varied, with participants finding it easier to carry out techniques if they had previous experience. Inability to carry out techniques appeared more common in the DVD group, apart from one participant in the face-to-face group who experienced severe problems with breath holding.

“ I have to say um, no matter how I tried, and on the DVD it said it would come eventually, I cannot breathe through my diaphragm.” (P7, female, DVD)

Some participants found it difficult to apply the techniques in particular situations:

“I can’t quite master the stomach breathing when I am moving around, but no doubt that will come.” (P3, female, DVD)

## Discussion

### Main findings

Reasons for taking part in the BREATHE trial included being asked to, enhance progress in research, feel better/reduce symptoms and reduce medication. Participants in the face-to-face group had a positive impression of the physiotherapist, liked receiving treatment tailored to their needs, and found meetings motivational. All participants liked the materials. Impact of breathing retraining included regular practice, leading to increased awareness of breathing and development of new habits. Perceived benefits of breathing retraining included increased control over breathing, reduced need for medication, feeling more relaxed, and improved health and QoL. However, problems included difficulties a) finding time to practice and b) mastering techniques.

### Interpretation of the findings in relation to previously published work

There was high motivation for engagement with breathing retraining. Participants wanted to improve their asthma control and reduce reliance on medication. Previous research has also shown that people with asthma adopt non-pharmacological methods of management to reduce reliance on medication.^5,14^ Most participants reported practising intensively (more than three times a day) for the initial 3–4 weeks, as recommended by the physiotherapist and in the booklet. Following this, participants reported increased awareness of their breathing, and that breathing techniques had become habitual, in line with research showing that habit formation is an effective health behaviour change strategy.^18^

Participants in the face-to-face group were very positive about the physiotherapist. Many said meetings motivated them to keep on with their exercises, and all participants in the DVD group said they would have preferred to see the physiotherapist. Similarly, in a trial of vestibular rehabilitation for dizziness delivered with or without telephone support, all participants reported preferring telephone support.^19^ Reported inability to master certain techniques appeared to be more common in the DVD group. While participants across both groups mentioned finding diaphragm breathing, breath holding and slow breathing difficult initially, several in the DVD group reported being unable to master diaphragm breathing. Those who saw the physiotherapist said she had tailored their treatment, and felt able to improve with this support and encouragement. However, the BREATHE trial showed that face-to-face physiotherapy was no more effective than the DVD (although both were superior to usual care) (submitted for publication). Similarly, in a trial of vestibular rehabilitation, telephone support did not lead to greater improvements than a booklet.^20^ While personal contact may enhance confidence in carrying out techniques, this does not necessarily lead to greater benefits. However, it is possible that a minority of individuals may need face-to-face instruction in order to master novel techniques.

Participants felt that breathing training brought about a variety of benefits. In particular, these included better asthma control (which meant participants were able to breathe their way through asthma attacks, in some cases possibly even avoiding hospital admissions) and reduced use of reliever medication (which meant participants felt more in control of their asthma, and less worried if inhalers were not available). Participants also reported feeling more relaxed, having more energy and sleeping and feeling better. Previous breathing retraining trials have found similar benefits.^12,15^

Many reported difficulty in finding time to do the breathing exercises, until they were able to make it part of their routine. One participant reported dropping out of the trial due to difficulty finding time to practice. People who saw the booklet during the development phase predicted it would be difficult for those working full time and/or with young children to find time to do it.^14^ However, all participants in this study saw the relevance of breathing retraining, possibly because the trial was open only to those whose QoL was affected by asthma.

### Strengths and limitations of the study

This is the first full paper of patients’ experiences of breathing retraining for asthma self-management. However, it has several limitations. Participants were randomised to their treatment arm of BREATHE, but self-selected into this qualitative study, so may not represent views of all who received the interventions. Despite attempts to reduce socially desirable responding, participants may have felt pressure to report positive outcomes. Also, the first author (E.A.C.), who conducted the interviews, had been involved in the development of the booklet and DVD used in BREATHE. On one hand, this dual involvement meant that E.A.C. had a good understanding of breathing retraining. On the other hand, this is a source of potential bias as it meant E.A.C. may have been (unconsciously) less open to criticism of breathing retraining.

### Implications for future research, policy and practice

Breathing retraining was well received by participants in both groups, and is likely to be acceptable and valued as an adjuvant treatment in general practice. Patients were positive about face-to-face physiotherapy due to liking the physiotherapist, appreciating that she tailored the content to their needs and finding appointments motivational. However, the DVD is much more widely available and costs considerably less to deliver. Further, face-to-face physiotherapy did not demonstrate increased benefit in terms of quality of life in this patient group. Therefore, making the breathing retraining DVD widely available may lead to improved asthma control in the general population. To increase the confidence of individuals carrying out breathing retraining delivered by DVD, it might be helpful to inform them that research has not shown greater benefits for face-to-face physiotherapy.

## Conclusions

Breathing retraining delivered face-to-face or by DVD plus booklet was acceptable and valued by almost all participants, with many reporting improved wellbeing. It is therefore likely to be well received as a method of asthma management.

## Method

### Design

This study followed a qualitative design, using semi-structured telephone interviews to explore participants’ experiences of breathing retraining for asthma delivered by DVD or face-to-face physiotherapy.

### Treatment arms

The DVD group were given the ‘Breathing Freely’ DVD and booklet. The DVD provides detailed demonstrations and explanations of how to carry out breathing techniques, with further information and support in the printed booklet. The face-to-face physiotherapy group received the same booklet (but not DVD) and had three one-to-one sessions lasting up to 1 h with a respiratory physiotherapist over a period of 4–6 weeks.

### Participants

Participants were eligible if they had taken part in one of the two intervention arms of the BREATHE trial (inclusion criteria for the trial are described in the BREATHE protocol paper).^16^ This pragmatic randomised trial recruited adults diagnosed and treated for asthma in the community who had sub-optimal control assessed by validated disease-specific symptom and QoL assessments. Initially, consecutive sampling of participants in the pilot trial was used. Purposive sampling was used towards the end to ensure adequate representation of male participants. Recruitment continued until the data reached saturation. The final sample comprised 11 women and 5 men between the ages of 23 and 70 (mean age 55.19, SD 12.87). There were eight participants (2 men) in the DVD group, and eight (3 men) in the face-to-face group.

### Data collection

This study was approved by the National Research Ethics Service. Methods were performed in accordance with relevant regulations and guidelines. Interviews were conducted by the first author (E.A.C.), who had previous experience of conducting qualitative interviews, and developed the booklet and DVD used in BREATHE, between February 2013 and April 2014.

Consent was obtained from all patients prior to the start of the study, after which E.A.C. contacted patients to confirm they were willing to take part and agree an appropriate time for a telephone interview. Before starting interviews, participants were informed that the study aimed to understand their experiences of the treatments they received, and it was emphasised there were no right or wrong answers. This aimed to reduce the likelihood of participants giving socially desirable responses, such as not wanting to mention negative aspects of their treatment. The interview schedule was developed by the first author (E.A.C.), based on a qualitative process interview being used in a similar study, and checked by S.K., who had experience in conducting qualitative process interviews. The interview schedule included broad, open-ended questions with follow-up prompts. Topics covered included expectations of breathing retraining, experience of the breathing retraining programme and practising techniques, any problems experienced, what participants liked about the programme, if anything, any concerns participants had experienced, potential changes participants had experienced in their day to day life, and any advice participants would give to others thinking of carrying out breathing retraining. Following the first couple of interviews, participants were asked how they felt about each technique. Interviews were tailored according to participants’ responses, to enable exploration of topics spontaneously raised by participants. They were audio recorded and transcribed verbatim by an independent administrator.

### Data analysis

Transcripts were analysed by E.A.C. using inductive thematic analysis.^21^ Transcripts were read carefully several times to ensure familiarity with the data. A coding manual was developed on the first few transcripts to ensure transparent and systematic coding of data, and frequently revised. Themes were continually compared with newly coded transcripts to ensure they applied to the data, and to identify further potential themes. The coding manual was then checked with L.Y. (co-author) and a sample of texts second coded, to ensure good inter-rater reliability. Themes were checked for differences as a function of group.

### Data availability

The findings that support this study are available from the corresponding author on reasonable request.

### Clinical trial registration

Breathing Retraining for Asthma Trial of Home Exercises (BREATHE) is registered at www.ISRCTN.org, trial registration number 88318003.

## References

[1] Rabe KF, Vermeire PA, Soriano JB, Maier WC (2000). Clinical management of asthma in 1999: the Asthma Insights and Reality in Europe (AIRE) study. Eur. Respir. J.

[2] Rabe KF, Vermeire PA, Soriano JB, Maier WC (2000). Clinical management of asthma in 1999: the Asthma Insights and Reality in Europe (AIRE) study. Eur. Respir. J.

[3] Demoly P, Annunziata K, Gubba E, Adamek L (2012). Repeated cross-sectional survey of patient-reported asthma control in Europe in the past 5 years. Eur. Respir. Rev.

[4] Bishop FL, Yardley L, Lewith GT (2008). Treat or treatment: A qualitative study analyzing patients’ use of complementary and alternative medicine. Am. J. Public Health.

[5] Brien SB (2011). Integrated medicine in the management of chronic illness: a qualitative study. Br. J. Gen. Pract.

[6] Slader CA (2006). Complementary and alternative medicine use in asthma: who is using what?. Respirology.

[7] Bowler SD, Green A, Mitchell CA (1998). Buteyko breathing techniques in asthma: a blinded randomised controlled trial. Med. J. Aust.

[8] Bruton A, Thomas M (2011). The role of breathing training in asthma management. Curr. Opin. Allergy. Clin. Immunol..

[9] Cooper S (2003). Effect of two breathing exercises (Buteyko and pranayama) in asthma: a randomised controlled trial. Thorax.

[10] McHugh P, Aitcheson F, Duncan B, Houghton F (2003). Buteyko breathing technique for asthma: an effective intervention. NZ Med. J.

[11] Opat AJ, Cohen MM, Bailey MJ, Abramson MJ (2000). A clinical trial of the Buteyko breathing technique in asthma as taught by a video. J. Asthma.

[12] Thomas M (2009). Breathing exercises for asthma: a randomised controlled trial. Thorax.

[13] Bott J (2009). Guidelines for the physiotherapy management of the adult, medical, spontaneously breathing patient. british thoracic society physiotherapy guideline development group. Thorax.

[14] Arden-Close E (2013). Patients’ perceptions of breathing training for asthma: a qualitative study. Prim. Care Respir. J..

[15] Slader CA (2006). Double blind randomised controlled trial of two different breathing techniques in the management of asthma. Thorax.

[16] Bruton A (2013). The BREATHE study: Breathing REtraining for Asthma – Trial of Home Exercises. A protocol summary of a randomised controlled trial. Prim. Care Respir. J..

[17] Yardley L, Ainsworth B, Arden-Close E, Muller I (2015). The person-based approach to enhancing the acceptability and feasibility of interventions. Pilot Feasibility Stud.

[18] Lally P, Chipperfield A, Wardle J (2008). Healthy habits: efficacy of simple advice on weight control based on a habit –formation model. Int. J. Obes..

[19] Muller I, Kirby S, Yardley L (2015). Understanding patient experiences of self-managing chronic dizziness: a qualitative study of booklet-based vestibular rehabilitation, with or without remote support. BMJ Open.

[20] Yardley L (2012). Clinical and cost effectiveness of booklet based vestibular rehabilitation for chronic dizziness in primary care: single blind, parallel group, pragmatic, randomised controlled trial. BMJ.

[21] Braun V, Clarke V (2006). Using thematic analysis in psychology. Qual. Res. Psychol..

